# A New Fractional Particle Swarm Optimization with Entropy Diversity Based Velocity for Reactive Power Planning

**DOI:** 10.3390/e22101112

**Published:** 2020-10-01

**Authors:** Muhammad Waleed Khan, Yasir Muhammad, Muhammad Asif Zahoor Raja, Farman Ullah, Naveed Ishtiaq Chaudhary, Yigang He

**Affiliations:** 1 Department of Electrical and Computer Engineering, COMSATS University Islamabad, Attock 22060, Pakistan; engrwaleedkhan37@gmail.com (M.W.K.); yasir.ee@ciit-attock.edu.pk (Y.M.); rajamaz@yuntech.edu.tw (M.A.Z.R.); farmankttk@ciit-attock.edu.pk (F.U.); 2 Future Technology Research Center, National Yunlin University of Science and Technology, 123 University Road, Section 3, Douliou, Yunlin 64002, Taiwan; 3 Department of Electrical Engineering, International Islamic University, Islamabad 44000, Pakistan; 4 School of Electrical Engineering and Automation, Wuhan University, Wuhan 430072, China

**Keywords:** optimization, fractional swarming, entropy metric, optimal power flow (OPF), reactive power planning (RPD), shannon entropy

## Abstract

Optimal Reactive Power Dispatch (ORPD) is the vital concern of network operators in the planning and management of electrical systems to reduce the real and reactive losses of the transmission and distribution system in order to augment the overall efficiency of the electrical network. The principle objective of the ORPD problem is to explore the best setting of decision variables such as rating of the shunt capacitors, output voltage of the generators and tap setting of the transformers in order to diminish the line loss, and improve the voltage profile index (VPI) and operating cost minimization of standard electrical systems while keeping the variables within the allowable limits. This research study demonstrates a compelling transformative approach for resolving ORPD problems faced by the operators through exploiting the strength of the meta-heuristic optimization model based on a new fractional swarming strategy, namely fractional order (FO)–particle swarm optimization (PSO), with consideration of the entropy metric in the velocity update mechanism. To perceive ORPD for standard 30 and 57-bus networks, the complex nonlinear objective functions, including minimization of the system, VPI improvement and operating cost minimization, are constructed with emphasis on efficacy enhancement of the overall electrical system. Assessment of the results show that the proposed FO-PSO with entropy metric performs better than the other state of the art algorithms by means of improvement in VPI, operating cost and line loss minimization. The statistical outcomes in terms of quantile–quantile illustrations, probability plots, cumulative distribution function, box plots, histograms and minimum fitness evaluation in a set of autonomous trials validate the capability of the proposed optimization scheme and exhibit sufficiency and also vigor in resolving ORPD problems.

## 1. Introduction

The Reactive Power Dispatch (RPD) problem, due to its extreme significance in contemporary load monitoring and management systems of power distribution companies, has been receiving mounting attention from power system engineering scholars with the goal of refining the system voltage profile index (VPI) and diminishing electrical system losses while keeping the constraints of system operation within allowed limits. RPD planning is an obligatory requirement for efficient and viable operation of the electrical transmission $\left(T_{line}\right)$ and distribution $\left(D_{line}\right)$ systems. Presently, the existing $T_{line}$ and $D_{line}$ systems are involuntary to operate at almost full capacity due to imbalance investment in transmission and distribution sector as well as power generation sector. More often, due above prevailing situation the heavy flows of current in entire system tend to incur losses as well as threatening stability of the electrical system [1]. This ultimately creates unenviably augmented risk of electricity outages in entire system of different severity levels. In this pertinent situation, there is a general consensus developed amongst the network operators to reinforce the existing transmission $\left(T_{line}\right)$ and distribution $\left(D_{line}\right)$ system by installation of new lines and power grid stations to make it efficient, smart, reliable and fault tolerant [2]. To meet the highlighted challenges, there are two potential options available which are normally exercised by different operators with certain allied merits and demerits. The first option is associated to augmentation of the existing infrastructure of electrical power system through the addition of new lines (i.e., LT and HT lines) and substations. The merits and demerits of this option are provided in Table 1.

The second option is related to utilization of the existing electrical transmission and distribution system by optimal setting of its performance parameters which resultantly improve the efficacy of entire electrical system. This can be achieved by carrying out specialized technical study of electrical system known as the optimal power flow (OPF). The merits and demerits of this option are provided in Table 2.

**Figure 1:**
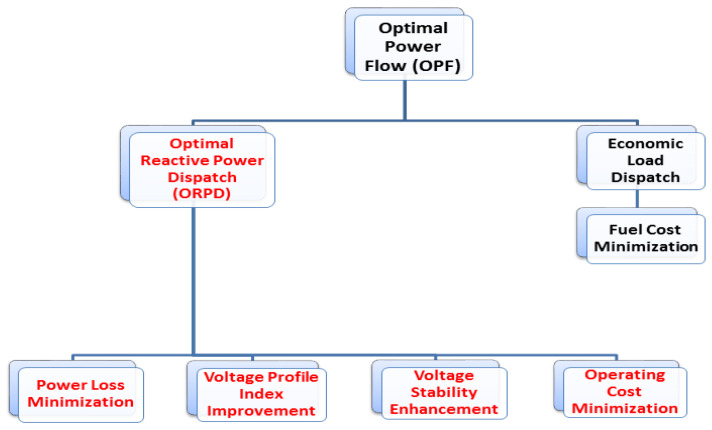
Sub problems of optimal power flow (OPF).

The OPF model is employed in an interconnected electrical system to set the operating parameters of power plants in such a way to efficiently meet the expected load dispatch demand of potential consumers with minimum operating cost and power losses. The OPF model is further divided into two sub problems in which first one is known as economic load dispatch and the other sub-problem is known as ORPD. Both problems are applied in different scenarios based on the requirement of operator subjected to get the desired objective functions. The sub problem of OPF are illustrated in Figure 1.

In this research proposal we have nominated the sub problem of OPF known as ORPD for addressing the objective function of electrical system such as $P_{loss}$ minimization, voltage profile index (VPI) improvement and operating cost minimization [3,4,5,6]. The ultimate goal of ORPD is to reduce the $T_{line}$ and $D_{line}$ losses and improve the voltage profile index (VPI) while keeping the decision variables of electrical system such as shunt capacitor banks $\left(Q_{C}\right)$, generator voltages $\left(V_{G}\right)$, and transformer taps (*T*) setting within the limits in standard power systems. The objective functions of the ORPD such as power loss minimization, VPI improvement and overall cost minimization are modeled as complex nonlinear equations. The solution of these equations using deterministic techniques (such as RK method, Adams method, shooting method and spectral analysis method etc.) is not possible. The problems of RPD have widely been solved using stochastic methods [6]. To eliminate the RPD problems, various optimization algorithms have been implemented over a period of time to achieve the optimal results. Some of the known stochastic methods implemented for solution of RPD problem includes the linear programming (LP) [7], interior point method (IPM) [8], quadratic programming (QP) [9], genetic algorithm (GA) [10], particle swarm optimization (PSO) [11,12], multi-objective optimization particle swarm optimization (MOPSO) algorithm [13], fractional Order PSO (FO-PSO) [6], harmony search algorithm (HSA) [14], gaussian bare-bones water cycle algorithm (NGBWCA) [15], tabu search (TS) [16], comprehensive learning particle swarm optimization [17], teaching learning based optimization (TLBO) [18], adaptive GA (AGA) [19], seeker optimization algorithm (SOA) [20], jaya algorithm [21], differential evolution (DE) [2,3,5,22,23,24,25], Artificial Bee Colony Algorithm [26], Hybrid Artificial Physics PSO [27], improved antlion optimization algorithm [28], Chaotic Bat Algorithm [29], classification-based Multi-objective evolutionary algorithm [30], evolution strategies (ES) [31], evolutionary programming (EP) [32], firefly algorithm (FA) [33], gravitation search optimization algorithm (GSA) [34,35], bacteria foraging optimization (BFO) [36], bio-geography-based optimization algorithm (BBO) [37] and grey wolf based optimizer algorithm (GWO) [38]. In 2017, another advanced optimizer has also been applied to the problems RPD known as gradient-based WCA (GWCA) [39,40] and results demonstrate the relevance and productivity of optimizer. Furthermore, in order to solve the non-linear complex problems of RPD in power system author Heidari et al. has proposed another state-of-the-art algorithm known as chaotic WCA (CWCA) in his research proposal [41].

These methods have their own merits and demerits in solving the problems of RPD, still certain complications are persisted due to multi modal, nonlinear and discrete characteristic of power system which needs to be catered in more appropriate manners. In addition, due to the complex non-linear and non-differential nature of the RPD problems, a wider set of employed optimization methods coverages towards sub optimal solutions. Presently many heuristic models have been applied under circumstances where normal schemes cannot find a satisfactory result in case of discontinuous and non-differential function with large number of nonlinearly related parameters [14,15]; however, the fractional dynamics of particle swarm optimization along with entropy diversity has not yet been explored in energy and power sector, specifically in solving ORPD problems.

**Figure 2:**
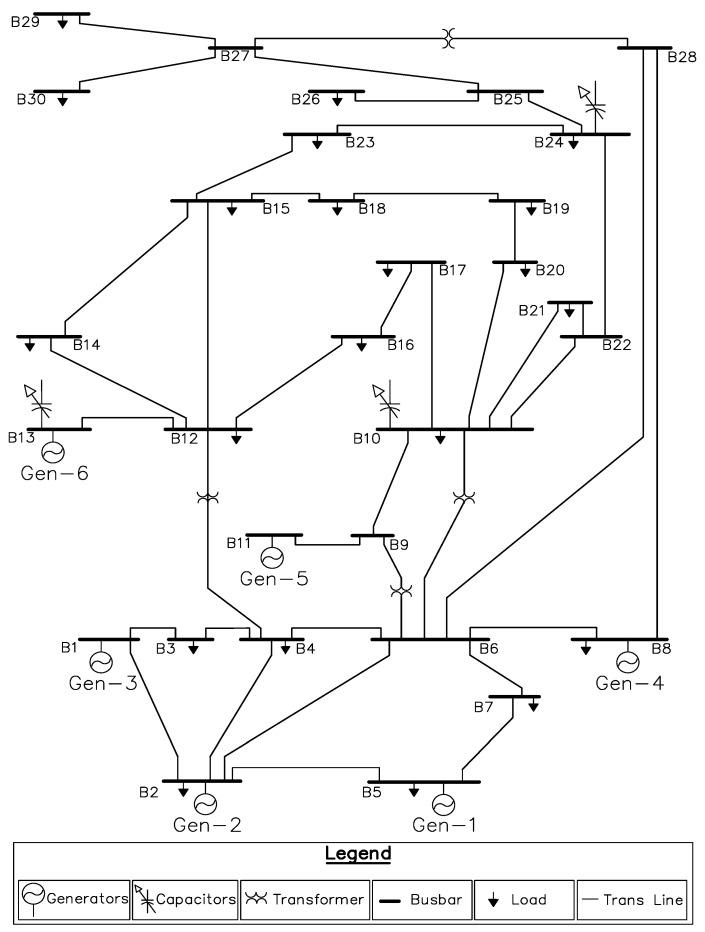
Thirty-standard bus system single-line diagram (SLD).

**Figure 3:**
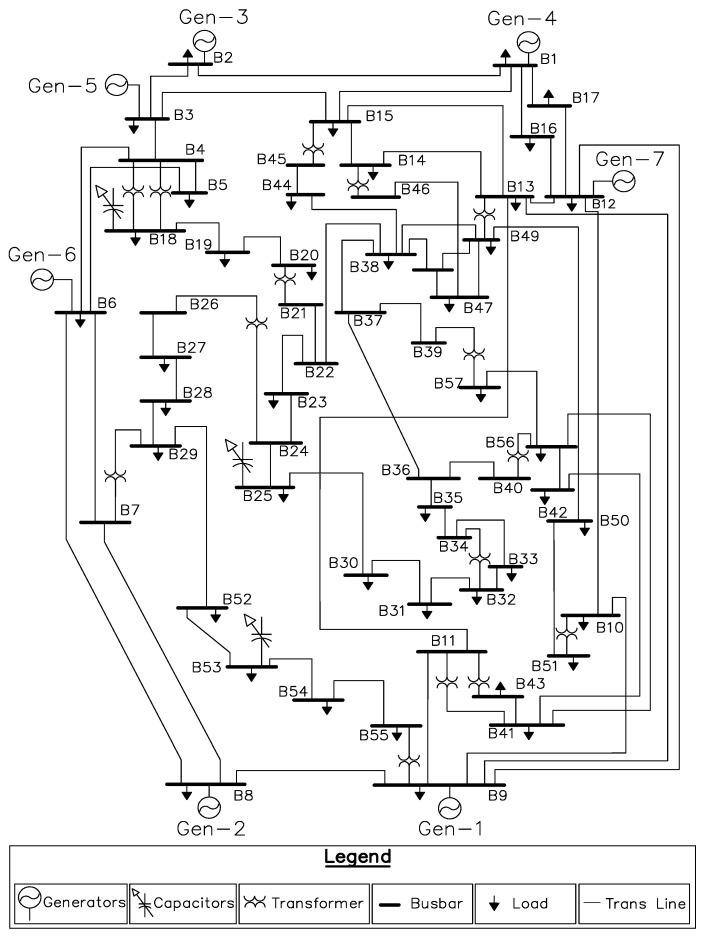
Fifty-seven-standard bus system SLD.

The concept of fractional PSO is based on incorporating the fractional derivative and underlying theory inside the mathematical model of conventional PSO to improve the convergence rate while the synergy of entropy metric improve the optimization characteristic of algorithm by avoiding the suboptimal solution. In recent years, the fractional calculus based optimization mechanisms have been effectively applied in domain of science and engineering such as feature selection [42], image processing and segmentation [43,44], swarm robotics [45], fuzzy controllers [46], classification of extreme learning machine [47], adaptive extended Kalman filtering [48], electromagnetics [49], hyperspectral images [50], media-adventitia border detection [51], monitoring [52] and fractional adaptive filters [53]. Similarly, the inclusion of entropy diversity inside the optimizer is found to be effective in enhancing its performance by means of avoiding premature convergence [54,55,56,57]. Keeping this in mind, in present study, a new algorithm, namely, fractional order particle swarm optimization (FO-PSO) with entropy metric, is designed with synergy of both the fractional calculus and Shanon entropy, to address RPD problem through assessment and proper tuning of decision variables including VAR compensator $\left(Q_{C}\right)$, setting of transformers tap (*T*) and generator voltage $\left(V_{G}\right)$ while meeting the objectives of system operators (i.e., consumers load demand and minimal losses in power system). The proposed optimization algorithm is tested on standard 30 and 57-bus systems. The single-line diagram (SLD) of the standard 30 and 57-bus test system is illustrated in Figure 2 and Figure 3.

The outcomes are compared with their counterpart algorithms to validate the capability of the proposed optimization scheme and exhibit sufficiency besides, vigor to resolve ORPD problems. The apotheosis of the contribution is stated below:

- Novel application of fractional evolutionary strategy with introduction of the velocity based entropy diversity in internal solver of the optimization algorithm for solving RPD problems in standard power systems.
- Effective application of designed scheme to improve the performance of power systems in terms of power loss $P_{loss}$ minimization, operating cost minimization and improving voltage profile index (VPI) while fulfilling the system load demands and operational constraints.
- The endorsement of the algorithm performance through outcomes of statistical analysis in terms of histogram studies, learning curves and probability charts which exhibit the accuracy, reliability, strength and constancy of the proposed optimization strategy.
- Flexibility in degree of freedom is acquirable for solving the optimization assignments by using the variants of FO-PSO based on fractional orders α=[0.1,0.2,…,0.9].
- The brilliance of FC along with entropy is exploited in optimization scenarios to design a substitute and feasible algorithm for problem in energy sector related to transmission and distribution segment.

This presented research article is aligned as follows: In Section 2, the mathematical model for ORPD is formulated. Section 3, describe the methodology of the designed optimization technique known as FO-PSO with entropy metric, along with graphical abstract and pseudo-code of presented optimizer. In Section 4, simulation outcomes and discussion is presented for the proposed technique along with comparative analysis through statistics, while the last section of paper summarizes the conclusions.

## 2. Problem Formulation

This section briefs the mathematical model of objectives functions including the $P_{loss}$ minimization, VPI improvement and overall cost minimization, as given below.

### 2.1. System $P_{loss}$ Minimization

The system $P_{loss}$ is calculated by means of the following expression [6,15,23].

(1) $$\begin{array}{c} A_{1}=P_{Loss}=\sum_{h\geq 1}^{ml}g_{h}\left[V_{a}^{2}\;-\;2\times V_{a}V_{b}\cos\left(\delta_{a}-\;\delta_{b}\right)+V_{b}^{2}\right] \end{array}$$

where ml represents the no. of transmission lines, $V_{a}$, $V_{b}$ and $\delta_{a}$, $\delta_{b}$ represents magnitude and phase angle of voltage at the end buses a and b of hth line and $g_{h}$ used to represent the conductance of the hth line of standard bus system. Data for standard IEEE (100 MVA base) 30-bus system is given in Table 3, Table 4, Table 5 and Table 6 for reference.

### 2.2. Improvement of Voltage Profile (VPI)

Voltage Profile Index (VPI) can be enhanced by limiting the load bus deviations from 1.0 per unit system. Mathematically, the fitness function of VPI is stated as [6,23].

(2) $$A_{2}=\;\sum_{j\epsilon \;ML}\left|V_{j}\;-\;1.00\right|$$

where ML represents the no. of load buses.

### 2.3. Operating Cost Minimization

This fitness function for operating cost minimization includes the cost due to energy loss in the power systems which can be expressed as:

(3) $$C_{total}=C_{Ener}=P_{loss}\times 0.06\times 1000\times 24\times 365$$

(4) $$C_{Ener}=P_{loss}\times 525,600$$

where we can fix the following values. Installation cost of shunt capacitor as per market study is approximately 1000 USD. Cost incurred in power system due to energy loss is approximately 0.067 USD/kWh. Furthermore, there is total 365 days in a year and 24 h in a single day.

### 2.4. System Constraints

System Constraints are divided into the following:

#### 2.4.1. Equality Constraints

The *P* and *Q* power balance relationships are adopted as equality constraints in ORPD studies. The mathematical representation of these expressions are formulated below:

(5) $$\begin{array}{c} -V_{a}\sum_{b=1}^{KB}V_{b}\left[G_{ab}\cos\left(\delta_{a}-\delta_{b}\right)+B_{ab}\sin\left(\delta_{a}-\delta_{b}\right)\right]-P_{Da}+P_{Ga}=0 \end{array}$$

(6) $$\begin{array}{c} -V_{a}\sum_{b=1}^{KB}V_{b}\left[G_{ab}\sin\left(\delta_{a}-\delta_{b}\right)-B_{ab}\cos\left(\delta_{a}-\delta_{b}\right)\right]-Q_{Da}+Q_{Ga}=0 \end{array}$$

where $G_{ab}$ and $B_{ab}$ denotes the standard bus system line conductance and susceptance between *a*th and *b*th bus. Moreover, a=1…,KB where KB represents the no. of system buses, $Q_{G}$ denotes the generator output *Q*, $P_{G}$ denotes the generated *P*, $Q_{D}$ represents the demanded *Q*, $P_{D}$ represent the demanded *P*.

#### 2.4.2. Inequality Constraints

It includes the following:

- Shunt Capacitor $\left(Q_{d}\right)$ limits, which are restricted by boundaries as follows:
  (7) $$Q_{dk}^{mi}\;\leq \;Q_{dk}\leq \;Q_{dk}^{mx}\;,\;k=\;1,\ldots ,\;M_{d}$$
- Transformer tap setting $\left(T_{k}\right)$, which are restricted by boundaries as follows:
  (8) $$T_{k}^{mi}\;\leq \;T_{k}\leq \;T_{k}^{mx}\;,\;k=\;1,\ldots ,\;MT$$
- Generator Voltages $\left(V_{G}\right)$ and output reactive power *Q* which are restricted by boundaries as follows:
  (9) $$V_{Gk}^{mi}\;\leq \;V_{Gk}\leq \;V_{Gk}^{mx}\;,\;k=\;1,\ldots ,\;MG$$
  (10) $$Q_{Gk}^{mi}\;\leq \;Q_{Gk}\leq \;Q_{Gk}^{mx}\;,\;k=\;1,\ldots ,\;MG$$

## 3. Methodology

**Figure 4:**
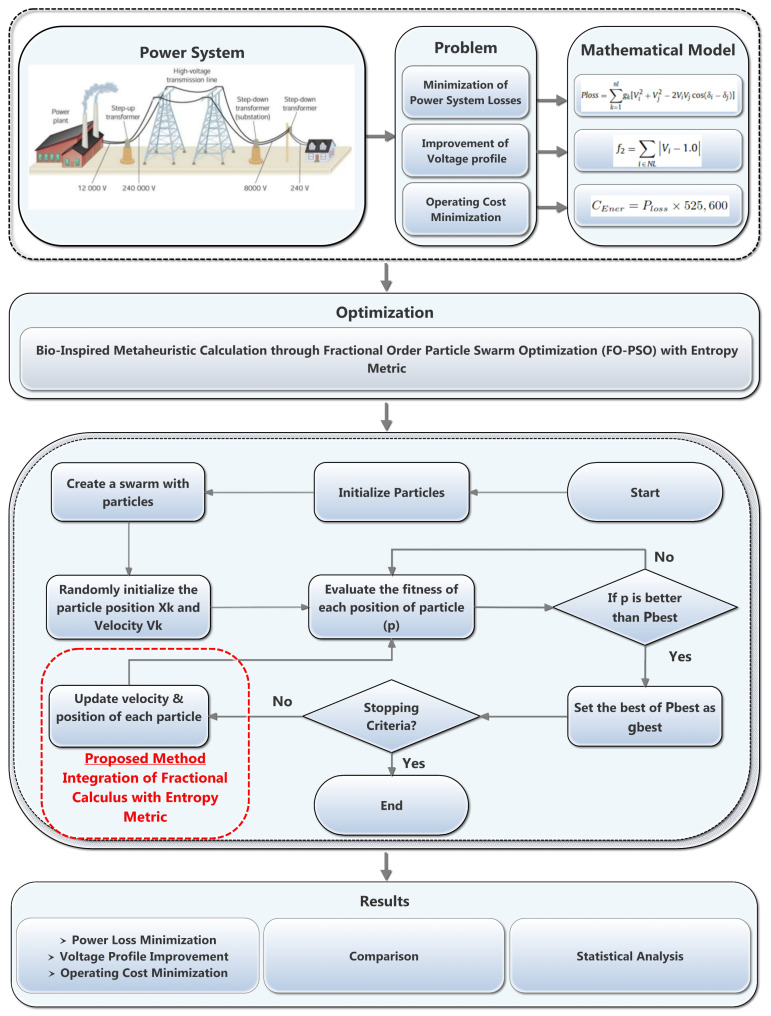
Overall design scheme for the proposed study.

The developed algorithm in the present study is based on FO-PSO with entropy metric to solve the optimization problems during RPD in standard 30 and 57-bus networks. This section is divided into two sub-sections; in first sub-section, a brief outline of proposed technique known as FO-PSO with entropy metric and its mathematical derivation is presented. Then, in second sub-section, the work flow of proposed optimization technique for solving ORPD problem along with pseudo-code and its allied clarifications are presented while the graphical abstract of the proposed scheme is shown as depicted in Figure 4.

### 3.1. Overview of FO-PSO with Entropy Metric

FO-PSO with entropy metric is a transformational stochastic model that extends PSO scheme [55,58,59,60,61] exploiting the function of typical selection in practical application, was established to improve the PSO limit in order to escape the local optima [15]. The proposed scheme has the capability to resolves variety of practical problems encountered by system operators in power systems due to its high solution quality and superior convergence rate characteristics then traditional model.

#### 3.1.1. Fractional PSO

Fractional calculus (FC) hypothesis is a useful tool for applied science [58,62,63,64,65] and has put considerable effort into enhancing the performance several applied algorithms used for filtering, identification, pattern recognition, reliability, controllability, observability and strength. In this research proposal, another optimization algorithm called fractional-order PSO with entropy metric computation is used to study the RPD problem.

In recent days the application of FC with stochastic function has opened new venues of research in power and engineering sector for specialists, e.g., power sector, fluid mechanics, engineering, and others. Mathematically, the FC can be derived in light of Grunewald-Letnikov definition which dependent on idea of fractional differential equation with fractional coefficient β∈ C of function *f* (*t*) is stated as:

(11) $$X^{\beta }\left[f\left(t\right)\right]=\lim_{J\to 0}\left[\frac{1}{J^{\beta }}\left(\sum_{m=0}^{+\infty }\frac{{\left(-1\right)}^{m}\gamma \left(\beta +1\right)f\left(t-mJ\right)}{\gamma \left(\beta -m+1\right)\gamma \left(m+1\right)}\right)\right]$$

where gamma function is denoted by γ [62]. The above stated equation possesses a significant property which suggests a finite arrangement known as integer-order derivative. Along these lines, whole number subsidiaries are “nearby” functions while fractional derivatives functions possess a “memory” of every previous occasion. However, after the elapse of certain time period the influence of past events diminishes. Based on Equation (11):

(12) $$X^{\beta }\left[f\left(t\right)\right]=\frac{1}{T_{s}^{\beta }}\sum_{m=0}^{Z}\frac{{\left(-1\right)}^{m}\gamma \left(\beta +1\right)f\left(t-mT\right)}{\gamma \left(m+1\right)\gamma \left(\beta -m+1\right)}\;$$

where sampling period of time is represented by $T_{s}$ and *Z* denotes the truncation order of function. This mathematical model is appropriate to depict phenomena of chaos and irreversibility because of its in-built memory property function.

Let assume the sampling period $T_{s}=1$ based on scientific work proposed in research article [58], the following mathematical expression of the function can be defined:

(13) $$\begin{array}{c} X^{\beta }\left[v_{t+1}^{m}\right]=\rho_{1}r_{1}\left({\overset{˘}{g}}_{t}^{m}-x_{t}^{m}\right)+\rho_{2}r_{2}\left({\overset{˘}{x}}_{t}^{m}-x_{t}^{m}\right)+\rho_{3}r_{3}\left({\overset{˘}{n}}_{t}^{m}-x_{t}^{m}\right) \end{array}$$

Comparable outcomes for r≥4 are obtained while performing initial test on the scheme proposed in the literature. Similarly, the mathematical prerequisites of the scheme proposed increment with *r* linearly. Subsequently, for *r* = 4 order Equations (12) and (13) will be revised as Equation (14):

(14) $$\begin{array}{c} v_{t+1}^{m}=\beta v_{t}^{m}+\left(0.5\times \beta v_{t-1}^{m}\right)+\left(0.1666\times \beta {\;\left(1-\beta \right)v}_{t-2}^{m}\right)+\left(0.04166\times \beta {\;\left(1-\beta \right)\left(2-\beta \right)v}_{t-3}^{m}\right)\\ +\rho_{1}r_{1}\left({\overset{˘}{g}}_{t}^{m}-x_{t}^{m}\right)+\rho_{2}r_{2}\left({\overset{˘}{x}}_{t}^{m}-x_{t}^{m}\right)+\rho_{3}r_{3}\left({\overset{˘}{n}}_{t}^{m}-x_{t}^{m}\right) \end{array}$$

The coefficients β, $\rho_{1}$, $\rho_{2}$ and $\rho_{3}$ allocate weights to the proposed scheme inertial influence, $p_{best}$ and the $g_{best}$ while updating the new velocity and position of the optimizer, respectively. Normally, the value of inertial influence is close to 1 (slightly less than one in most cases). Based on the various characteristics of the scheme and the application of the problem, optimal setting of these parameters will improve the outcome of the proposed model. Furthermore, the parameters $r_{1}$, $r_{2}$ and $r_{3}$ represents the random vectors generally between 0 and 1.

From Equation (14), FO-PSO will be considered similar to a specific instance of the traditional PSO when fractional coefficient β=1 (without “memory”). Despite the fact that this new model joins the idea of FC, the difficulty to comprehend the fractional coefficient β influence still remains. Defined in literature [61,66], the behavior of the swarm can be separated in two exercises: First is related to abuse and second is related to investigation. The highlighted first exercise is related to convergence of the scheme while permitting a decent transient exhibition. However, in the event when the level of exploitation is excessively high, at that point the optimization model has the chance to get stuck on local outcome. The subsequent one translates the diversification of the model, while permitting the investigation of new solutions, resulting in the overall improvement of the scheme performance. However, the optimization model FO-PSO will take too much time and effort during the processing to find the best global result if the scheme level of exploration is excessively high.

The major trade-off between both the exploration and exploitation in traditional PSO has ordinarily been taken care by altering the inertial weight of the associated swarm which is also being exhibited by the Eberhart and Shi in his research article [67]. Furthermore, to enhance the activity of exploration an enormous inertial weight can be used while for exploitation the inertial weight should be minimal in order to get the best possible results. Since FO-PSO control and improve the convergence rate of the swarm through integration of FC concept with traditional PSO. Therefore, to get the best global outcome from the scheme significant level of the investigation has been carried out to define the fractional coefficient β.

This stochastic scheme is not much explored in the field of power sector specially in case of RPD problem. However, few researchers have employed this technique recently to RPD problem to get promising results [6,23] but the concept of entropy metric utilized in the internal solver of the model is not yet explored. So, we are going to propose this concept in order to get the advantage of both the techniques by improve the convergence rate of scheme and also discourages the premature convergence phenomenon encountered in some cases.

The concept of entropy with FO-PSO with entropy metric will be new domain for various researcher in the field of power and applied engineering sector specifically to address RPD problem to get the optimal results.

#### 3.1.2. Entropy

Entropy is defined as quantitative measure of system transformation from ordered to disorder state [68,69,70]. Various explanations have been proposed consistently for entropy over the years some of which are state as freedom, chaos, measure of disorder, issue, mixing, tumult, spreading, opportunity and information data [71]. This concept is widely utilized in field of statistical mechanics, engineering and thermodynamics; however it describes the amount of data transmitted in a signal or activity performed. An instinctive understanding of the entropy with pre-defined distribution of probability relates to the amount of ambiguity about an event performed. In order to measure information loss in any event or activity a renowned information hypothesis developed by Shannon has been adopted by researchers as a reference in order to carry forward their investigation [55].

The essential depiction of entropy was proposed by Boltzmann to portray various transition of systems states. The phenomenon of spreading was implemented by Guggenheim to exhibit scattering imperativeness structure framework. Shannon portrayed H [72] as an extent of choice, and information:

(15) $$H\left(J\right)=-M_{c}\sum_{j\epsilon J}p_{i}\left(j\right)\log p_{i}\left(j\right)$$

The parameter $M_{c}$ is a positive constant which is normally equal to 1. Moreover, probability distribution p(k) is termed as a discrete random variable $\left\{j\epsilon J\right\}$. This can also be easily extended to random multi variables. For two variables $\left(j,k\right)\epsilon \left(J,K\right)$ entropy is defined as:

(16) $$H\left(J,K\right)=-M_{c}\sum_{j\epsilon J}\;\sum_{k\epsilon K}p_{i}\left(j,k\right)\log p_{i}\left(j,k\right)$$

**Figure 5:**
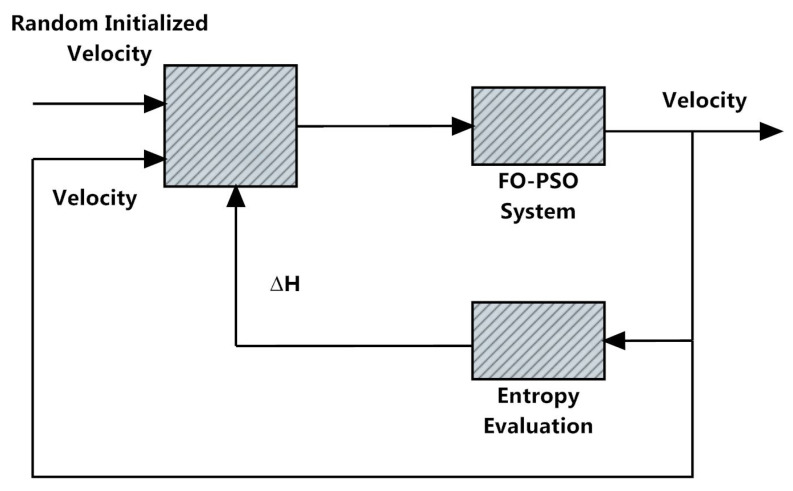
Fractional order (FO)–particle swarm optimization (PSO) with entropy with velocity reinitialization [73].

The introduction of entropy concept in FO-PSO algorithm will improve the optimizer rate of convergence as well as also avoiding premature convergence. In a second phase, the behavior of optimization technique FO-PSO is being influenced by the entropy signal, namely the updation of the velocity as shown in Figure 5, along the implementation for improving its rate of convergence.

Certainly, entropy exhibit the disorder of a employed system, i.e., a gauge of how speed up particles are within a system. Keeping in view, it is considered the velocity $v_{x}$ of any swarm particle and the velocity of best global particle. Each probability $p_{x}$, is given by the velocity of particle *x* to the velocity of best global particle over the maximum possible velocity. Therefore, the probability is expressed as:

(17) $$p_{x}=\frac{v_{x}}{v_{mx}}$$

where $v_{mx}$ denotes the maximum possible velocity for a particle. The Shannon particle diversity index, for a *n* swarm size, is based in quantification process and can be represented as:

(18) $$H\left(J\right)=-\sum_{j=1}p_{i}\left(j\right)\log p_{i}\left(j\right)$$

Entropy integrated with FO-PSO is proposed to promote the evaluation process of the model towards the best possible global optima. Comparison with several schemes have been undertaken to observe the performance of proposed model. The core motivation behind the use of entropy metric with FO-PSO is to improve the velocity updating mechanism of traditional algorithm by enhancing the convergence rate in order to attain the best possible optimal outcomes.

### 3.2. Application of FO-PSO with Entropy Metric in ORPD

The key variation in proposed optimization technique known as FO-PSO with entropy from traditional PSO is the utilization of fractional derivative component along with introduction of entropy in the internal solver of algorithm to update swarm velocity. In the proposed mechanism, the computational efficacy of FO-PSO with entropy metric is proved by solving the ORPD problems in 30 and 57 Bus networks by minimizing the power losses of system, improving the voltage profile index (VPI) and minimizing the operating cost of power system. The application of FO-PSO with entropy metric by means of process stages is illustrated in Algorithm 1.
**Algorithm 1** Pseudo-code for FO-PSO with entropy metric to solve Optimal Reactive Power Dispatch (ORPD) problem. 1:**procedure**In steps with input and outputs 2:  3:**Inputs**: Set swarm particles size, function iterations, bus, branch and generator data for IEEE Standard Test Bus System i.e., 30 Bus system with 13 control variables and 57-Bus system with 25 control variables 4:  5:**Objective Functions**: The objective functions of this study are:Power Loss MinimizationImprovement of Voltage Profile Index (VPI)Operating Cost Minimization 6:**Output**: Minimum loss of power as define in Equation (1), improved Voltage Profile Index(VPI) as define in Equation (2) and minimum operating cost as defined in Equation (3) 7:  8:**Start FO-PSO with Entropy Metric** 9:  10:**Step 1**: **Initialization**: Randomly generated initial swarm A, known as particle in n-dimensional search space:
(19)$$A\;=\;[T_{x1},\;T_{x2},\;T_{nt},\;V_{x1},\;V_{x2},\;\ldots V_{nv},\;Q_{x1},\;Q_{x2}\ldots Q_{nq}]$$Each swarm particle comprises of equal entries as no. of decision variables in the IEEE standard bus systems, which are to be optimized.Randomly initialize velocity $V_{k}$ and position $X_{k}$The entry of swarm is based on random generation within the defined lower and upper limit of independent decision variable of data set. Mathematically ith member of swarm is set as:
(20)$$A_{i,j}\left(0\right)=\left(A_{j}^{v}-A_{j}^{v}\right)\times rand\left(0,1\right)+A_{j}^{L}$$
where, rand(0,1) demonstrates the pseudo-random real numbers between 0 and 1. Setting parameters of proposed optimization algorithm is mentioned in Table 7 and Table 8 11:  12:**Step 2**: **Fitness evaluation**: Evaluate the fitness of each particle (P) of the swarm using Equation (1) for system $P_{loss}$ minimization, Equation (2) for improvement of voltage profile (VPI) and Equation (3) for operating cost minimization 13:  14:**Step 3**: **Termination Criterion**: Stopping criteria of the proposed algorithm is based on the following factors:Tolerance limit attains, i.e., predefine difference between current and previous best attainedOn execution of Total number of set iterations i.e., i=1,2…, 100If the algorithm exceeds the predefine limits of control variables
Go to **Step 5** if termination criteria is satisfied 15:  16:**Step 4**: **Algorithm Updating Mechanism**: FO-PSO with entropy metric algorithms is updated on the basis of two mechanisms that are particle position using Equation (21) as:
(21)x(n,j+1)=x(n,j+1)+x(n,j)
and updating velocity using Equation (22) as:
(22)$$\begin{array}{c} v_{t+1}^{m}=\beta v_{t}^{m}+\left(0.5\times \beta v_{t-1}^{m}\right)+\left(0.1666\times \beta {\;\left(1-\beta \right)v}_{t-2}^{m}\right)+\left(0.04166\times \beta {\;\left(1-\beta \right)\left(2-\beta \right)v}_{t-3}^{m}\right)\\ +\rho_{1}r_{1}\left({\overset{˘}{g}}_{t}^{m}-x_{t}^{m}\right)+\rho_{2}r_{2}\left({\overset{˘}{x}}_{t}^{m}-x_{t}^{m}\right)+\rho_{3}r_{3}\left({\overset{˘}{n}}_{t}^{m}-x_{t}^{m}\right) \end{array}$$
The coefficients β, $\rho_{1}$, $\rho_{2}$ and $\rho_{3}$ allocate weights to the proposed scheme inertial influence, $p_{best}$ and the $g_{best}$ while updating the new velocity and position of the optimizer, respectively. Update global best and local best for each particle and go to **Step 2** 17:  18:**Step 5**: **Storage**: Store the parameter of $g_{best}$ particle on the basis of objective functions 19:  20:**Step 6**: **Analysis**: Repeat **Steps 1 to 5** for different value of fractional order α in the algorithm for detailed statistical analysis of the results 21:  22:**Step 7**: **Replication**: Repeat the **Steps 1 to 6** for 30 an 57 bus networks 23:  24:**End of FO-PSO with Entropy Metric** 25:  26:**Step 8**: **Statistics**: Repeat the algorithm from **Steps 1 to 7** for several autonomous trials in order to analyze detailed performance of FO-PSO with entropy metric for optimal RPD systems

## 4. Simulation Results and Discussion

To validate the capability and efficacy of the proposed optimization technique known as FO-PSO with entropy metric, it has been tried and tested for problem of ORPD in standard 30 and 57 Bus networks with 13 and 25 variables. The FO-PSO with entropy metric method have been employed in MATLAB R2017b and the simulations are being performed on a PC core i5 having 8 GB RAM. The swarm size for the proposed technique is set to be 20 and 13 in case of standard 30 and 57 bus networks, respectively. The parameter settings used during the execution of algorithm evolution for 30 and 57 bus networks are listed in Table 7 and Table 8 accordingly.

**Table 7:** FO-PSO with entropy metric parameter settings of 30-bus system.

Parameters	Parameters Setting Value of Standard 30 Bus System (13 Variables)		
	Minimization of $P_{\mathrm{loss}}$	Improvement of VPI	Operating Cost Minimization
Fractional Order	0.3	0.4	0.7
Vmx	2.04	2.04	2.04
Factor of Local Acceleration	0.90–0.10	0.90–0.10	0.90–0.10
Factor of Global Acceleration	0.10–0.90	0.10–0.90	0.10–0.90
Inertia Weight	0.90–0.20	0.90–0.20	0.90–0.20
Particle Size or Decision Variables	13	13	13
Iterations During Statistics	60	30	80
Swarm Size	20	20	20

**Table 8:** FO-PSO with entropy metric parameter settings of 57-bus system.

Parameters	Parameters Setting Value of Standard 57 Bus System (25 Variables)		
	Minimization of $P_{\mathrm{loss}}$	Improvement of VPI	Operating Cost Minimization
Fractional Order	0.9	0.3	0.7
Vmx	2.04	2.04	2.04
Factor of Local Acceleration	0.90–0.10	0.90–0.10	0.90–0.10
Factor of Global Acceleration	0.10–0.90	0.10–0.90	0.10–0.90
Inertia Weight	0.90–0.20	0.90–0.20	0.90–0.20
Particle Size or Decision Variables	25	25	25
Iterations During Statistics	60	30	100
Swarm Size	13	13	5

The outcomes of the proposed optimizer technique is demonstrated in the relevant results tables in order to depicts the strength, capabilities and reliability in comparison to other state of the art techniques. The results obtained after implementation of FO-PSO with entropy metric technique in case of 30 bus and 57 bus system are duly compared with other optimization techniques results that have been directly taken from corresponding research publications of ORPD problems. Furthermore, the lower and upper limits of control variables are duly listed in Table 9.

### 4.1. ORPD for Standard 30-Bus System with 13 Variables

For contextual analysis, total 13 control variables of 30 bus system need to be optimized which includes four tap changer transformers (*T*), three compensator devices $\left(Q_{c}\right)$, and six voltages of generator $\left(V_{G}\right)$. To evaluate total actual losses incurred in power system, the proposed optimization algorithm is employed for ORPD in standard 30 bus system with 13 variables. The single line diagram of the same system has been depicted as Figure 2. The obtained outcomes are then duly compared and analyzed with similar problems reported outcomes of literature. The decision variables reported in these research studies of ORPD problems are delineated into the similar mathematical regime of MATPOWER [6,74] for a purpose to validate impartial comparison of the compared optimization techniques.

**Table 9:** Limits of control variables [38,75].

Control or Decision Variables	Lower Limit	Upper Limit
**IEEE 30-Test Bus System**		
Shunt VAR Compensators (MVar)	−12	36
Generator Voltages (p.u)	0.9	1.1
Setting of Transformer Tap Changer (p.u)	0.950	1.050
**IEEE 57-Test Bus System**		
Shunt VAR Compensators (MVar)	0	20
Generator Voltages (p.u)	0.94	1.06
Setting of Transformer Tap Changer (p.u)	0.900	1.100

Attained actual value of power system losses after implementation of FO-PSO with entropy metric algorithm is compiled in Table 10 along with reported values of power losses from other algorithms including PSO [76], HSA [14], GA [17], IWO [18], SGA [77], ICA [78], MICA-IWO [78], DE [23,79], R-DE [80], C-PSO [80], SFLA [81] and PSO-TVAC [82]. For effective assessment, all the compiled outcomes of line losses in 30 bus system with 13 decision variables in Table 10 are being examined using MATPOWER load flow.

It was observed that actual losses incurred by applying FO-PSO with entropy metric algorithm in case of 30 bus system are 4.628 MW, found to be 18.28% curtailed than the initial value while in contrast to the counterparts, the DE, HSA, GA, C-PSO, R-DE, GA, IWO, MICA-IWO, ICA, SFLA and PSO-TVAC provides 13.68%, 9.78%, 17.35%, 17.58%, 13.87%, 13.12%, 14.42%, 14.37%, 17.25% and 18.23% respectively as shown in Table 11.

**Figure 6:**
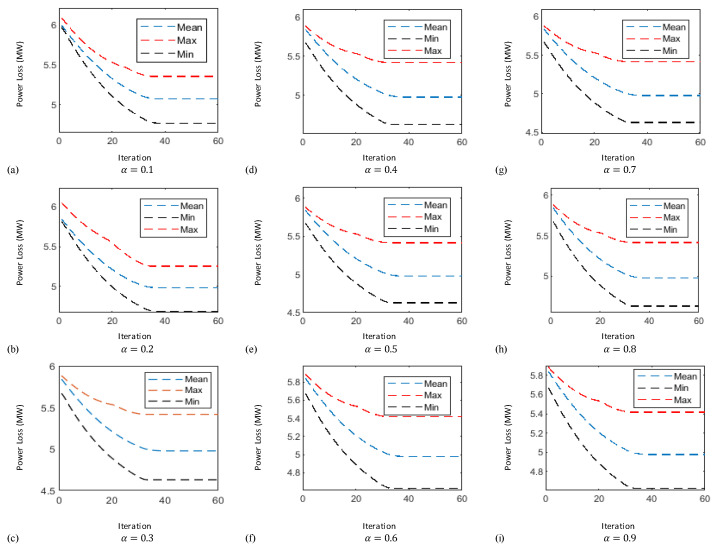
Thirty-bus system power loss (MW) minimization learning curves for (**a**) α = 0.1 (**b**) α = 0.2 (**c**) α = 0.3 (**d**) α = 0.4 (**e**) α = 0.5 (**f**) α = 0.6 (**g**) α = 0.7 (**h**) α = 0.8 (**i**) α = 0.9.

**Figure 7:**
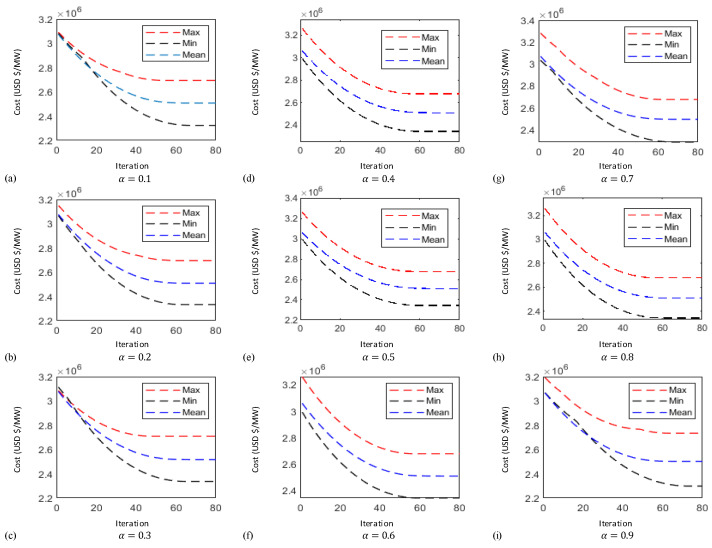
Thirty-bus system cost function minimization learning curves for (**a**) α = 0.1 (**b**) α = 0.2 (**c**) α = 0.3 (**d**) α = 0.4 (**e**) α = 0.5 (**f**) α = 0.6 (**g**) α = 0.7 (**h**) α = 0.8 (**i**) α = 0.9.

**Figure 8:**
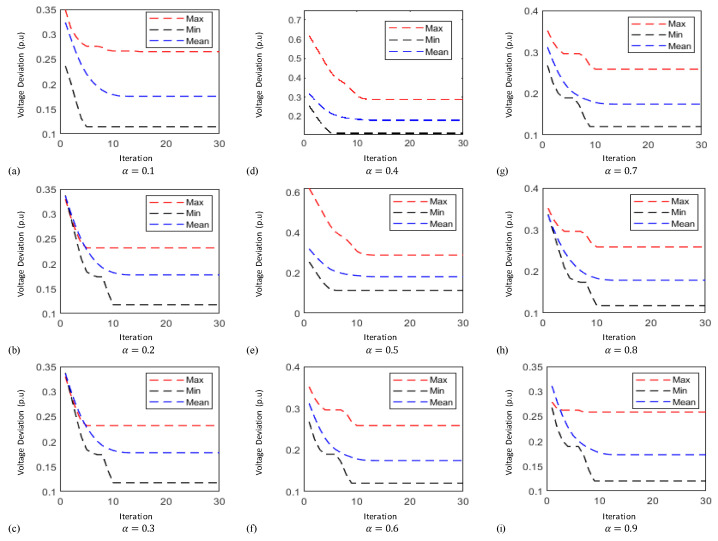
Thirty-bus system voltage profile index minimization learning curves for (**a**) α = 0.1 (**b**) α = 0.2 (**c**) α = 0.3 (**d**) α = 0.4 (**e**) α = 0.5 (**f**) α = 0.6 (**g**) α = 0.7 (**h**) α = 0.8 (**i**) α = 0.9.

In this case the results obtained by implementing the proposed technique known as FO-PSO with entropy metric stipulates preferable outcomes in tackling the ORPD problems and also remained within their identified boundaries. The learning curves of 30 bus test system are plotted in Figure 6, Figure 7 and Figure 8 showing the best, average and worst iterative updates of fitness function i.e., minimization of Power Losses, Cost Function and Voltage Profile Index (VPI) during 100 independent trials for α = 0.1, …, 0.9. Moreover, efficacy of proposed optimization algorithm can be evaluated by observing the learning power curves of fitness functions where the best results are obtained at fractional order α = 0.3 in case of power loss, α = 0.4 in case of voltage profile index improvement and α = 0.7 in case of operating cost minimization functions as shown in Figure 6, Figure 7 and Figure 8.

The proposed technique is also tested for fitness function of voltage profile improvement of power system and outcomes of simulation depicts that designed optimizer is considerably effective than other methods. The results achieved by the FO-PSO with entropy metric technique and some other state of the art algorithms such as SGA [77], PSO [77], NGBWCA [15], HSA [77], FA [33], BFOA [33], GWO [77], MFO [77] and OGSA [15] have been tabulated in Table 12.

**Table 10:** Comparison of optimized variable for 30-bus system with 13 decision variables.

Decision Variables	Published Outcomes												Present
	PSO [77]	HSA [14]	GA [17]	IWO [18]	SGA [77]	ICA [78]	MICA-IWO [78]	DE [23,79]	R-DE [80]	C-PSO [80]	SFLA [81]	PSO- TVAC [82]	FO-PSO with Entropy
**Transformer Tap Ratio (T)**													
**T6-9**	0.970	1.010	1.022	1.050	0.950	1.080	1.030	1.018	1.050	0.990	0.984	0.975	1.022
**T6-10**	1.020	1.000	0.991	0.960	0.980	0.950	0.990	0.979	0.900	1.050	1.020	0.927	1.046
**T4-12**	1.010	0.990	0.996	0.970	1.040	1.000	1.000	0.977	1.000	0.990	0.987	0.999	1.015
**T27-28**	0.990	0.970	0.971	0.970	1.020	0.970	0.980	1.009	0.970	0.960	1.008	0.964	1.011
**Generator Voltages (Vg)**													
**V1**	1.031	1.072	1.072	1.069	1.100	1.078	1.079	1.095	1.100	1.100	1.095	1.097	1.103
**V2**	1.011	1.062	1.063	1.060	1.042	1.069	1.070	1.085	1.094	1.100	1.091	1.087	1.101
**V5**	1.022	1.039	1.037	1.036	1.032	1.069	1.048	1.062	1.070	1.074	1.079	1.066	1.076
**V8**	1.003	1.042	1.044	1.038	0.981	1.047	1.048	1.065	1.073	1.086	1.070	1.070	1.080
**V11**	0.974	1.031	1.013	1.029	0.976	1.034	1.075	1.026	1.065	1.100	1.084	1.067	1.1083
**V13**	0.998	1.068	1.089	1.055	1.100	1.071	1.070	1.014	1.096	1.100	1.099	1.099	1.1076
**Capacitor Banks (Qc)**													
**Qc3**	17	34	5.350	8	12	−6	−7	20.223	10	9	NP	NP	4.7645
**Qc10**	13	12	36	35	−10	36	23	9.5843	0.26	0.30	3.965	1.0303	4.182
**Qc24**	23	10	12.417	11	30	11	12	13.029	0.12	8	4.205	4.653	11.458
**Ploss**	5.881	5.109	4.877	4.92	6.531	4.849	4.846	4.888	4.667	4.680	4.686	4.648	4.628

Note: NP stands for Not Provided.

**Table 11:** % Power loss reduction in standard 30-bus system with 13 decision variables.

Items	Base Case	DE [23,79]	HSA [14]	C-PSO [80]	R-DE [80]	GA [17]	IWO [18]	MICA-IWO [78]	ICA [78]	SFLA [81]	PSO-TVAC [82]	FO-PSO with Entropy
**Ploss (MW)**	5.663	4.888	5.109	4.680	4.667	4.877	4.920	4.846	4.849	4.686	4.648	4.628
**Loss reduction (%)**	-	13.680	9.780	17.350	17.580	13.870	13.120	14.420	14.370	17.250	18.23	18.280

**Table 12:** Comparison of voltage profile index (VPI) for standard 30-bus system with 13 decision variables.

Items	Published Outcomes									Present
	SGA [77]	PSO [77]	NGBWCA [15]	HSA [77]	FA [33]	BFOA [33]	GWO [77]	MFO [77]	OGSA [15]	FO-PSO with Entropy
**VPI (p.u)**	0.1501	0.1424	0.4773	0.1349	0.1157	0.1490	0.1260	0.1215	0.8085	0.1131

### 4.2. ORPD for Standard 57-Bus System Having 25 Variables

In this section, Standard 57-bus system with 25 decision variables system is being utilized to evaluate the efficacy of proposed technique known as FO-PSO with entropy metric. This ORPD problem will be handled as a search space of 25-dimension having 7 generator voltages (at buses B1, B2, B3, B6, B8, B9 and B12), 3 reactive power sources (at buses B18, B25 and B53) and 15 transformer taps. The single line diagram of the same system has been depicted as Figure 3. The optimization variables are obtained implementation of proposed technique in case of 57 bus system and then outcomes are collated with already established algorithms while keeping the *Q* and voltage constraints within the allowed limits.

Moreover, all the constraints of *Q* and voltage are scrutinized and not being violated. The corresponding outcomes obtained from simulations are recorded in Table 13 along with the previously published outcomes of other techniques in case of ORPD.

The attained value of $P_{loss}$ = 26.3956 MW after employment of proposed optimization algorithm which is 5.268% lower than the initial value and demonstrates a good undertaking of ORPD problem while in contrast to other counterparts such that PSO, ICA, ICA-PSO hybrid, FO-DPSO and DSA that provides 0.077%, 2.651%, 2.399%, 4.248% and 0.587% respectively, as illustrated in Table 14. In this case the results obtained by implementing the proposed technique stipulates preferable outcomes in tackling the ORPD problems and also remained within their identified boundaries.

**Figure 9:**
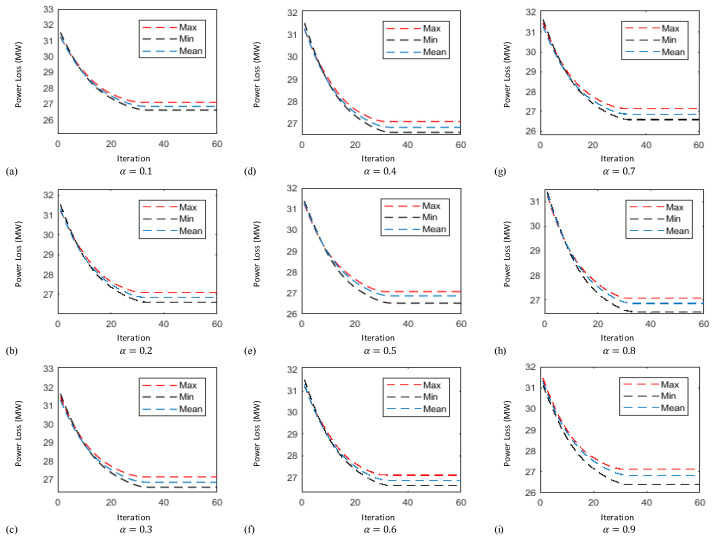
Fifty-seven-bus system Power Loss (MW) curves for (**a**) α = 0.1 (**b**) α = 0.2 (**c**) α = 0.3 (**d**) α = 0.4 (**e**) α = 0.5 (**f**) α = 0.6 (**g**) α = 0.7 (**h**) α = 0.8 (**i**) α = 0.9.

**Figure 10:**
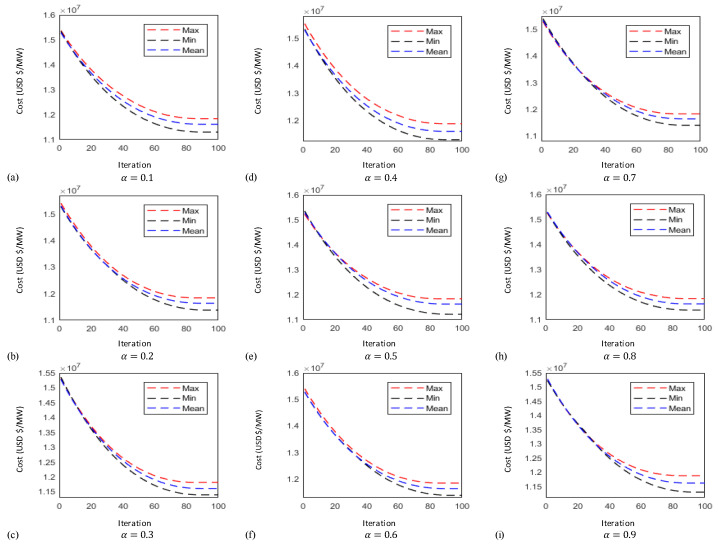
Fifty-seven-bus system cost function curves for (**a**) α = 0.1 (**b**) α = 0.2 (**c**) α = 0.3 (**d**) α = 0.4 (**e**) α = 0.5 (**f**) α = 0.6 (**g**) α = 0.7 (**h**) α = 0.8 (**i**) α = 0.9.

**Figure 11:**
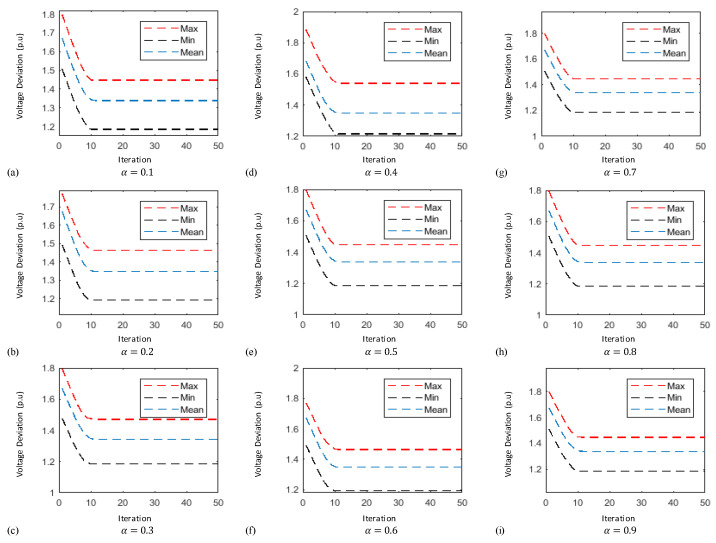
Fifty-seven-bus system voltage profile index curves for (**a**) α = 0.1 (**b**) α = 0.2 (**c**) α = 0.3 (**d**) α = 0.4 (**e**) α = 0.5 (**f**) α = 0.6 (**g**) α = 0.7 (**h**) α = 0.8 (**i**) α = 0.9.

In this case the results obtained by implementing the proposed technique stipulates preferable outcomes in tackling the ORPD problems and also remained within their identified boundaries. Moreover, the results of the simulation performed suggests by observing the learning power curves of fitness functions where the best results are obtained at fractional order α = 0.9 in case of power loss, α = 0.3 in case of voltage profile index improvement and α = 0.7 in case of operating cost minimization functions as shown in Figure 9, Figure 10 and Figure 11. Hence, the efficacy and computational distinction of proposed optimization scheme in case of undertaking the ORPD problem over the counterparts is clear from the comparative analyses.

**Table 13:** Comparison of optimized variable for 57-bus system with 25 decision variables.

Decision Variables	Published Outcomes								Present
	PSO [75]	OGSA [15]	WCA [15]	ICA [75]	Hybrid [75]	NGBWCA [15]	FO-DPSO [6]	DSA [83]	FO-PSO with Entropy
**Transformer Tap Ratio (T)**									
**T4-18**	0.97543	0.9833	1.0217	0.9584	0.9265	1.0185	0.9	0.9480	1.0459
**T4-18**	0.9716	0.9503	0.9614	0.9309	0.9532	0.9601	0.9209	1.0230	1.0252
**T21-20**	1.0286	0.9523	0.9496	1.0269	1.0165	0.9458	1.0268	1.0210	1.0230
**T24-26**	1.0183	1.0036	0.9901	1.0085	1.0071	0.9919	1.0075	0.9660	1.0231
**T7-29**	0.9401	0.9778	0.9986	0.9	0.9414	0.9951	0.9070	0.9270	1.0289
**T34-32**	0.94	0.9146	0.9000	0.9872	0.9555	0.9000	0.9871	0.9000	1.0415
**T11-41**	0.9761	0.9454	0.9634	0.9097	0.9032	0.9622	0.9010	0.9000	1.0100
**T15-45**	0.9211	0.9265	0.9063	0.9377	0.9356	0.9058	0.9	0.9770	1.0213
**T14-46**	0.9165	0.9960	0.9801	0.9166	0.9172	0.9764	0.9	0.9920	1.0283
**T10-51**	0.9044	1.0386	1.0631	0.9057	0.9337	1.0600	0.9165	0.9000	1.0094
**T13-49**	0.9118	0.9060	0.9131	0.9	0.9	0.9100	0.9	0.9530	1.0359
**T11-43**	0.92	0.9234	0.9294	0.9	0.9206	0.9302	0.9	0.9530	1.0302
**T40-56**	0.9891	0.9871	0.9782	0.9575	1.0042	0.9770	0.9980	1.0160	1.0315
**T39-57**	0.943	1.0132	1.0286	1.0476	1.0297	1.0271	0.9945	0.9000	1.0395
**T9-55**	0.9998	0.9372	0.9053	0.9	0.9294	0.9000	0.9	0.9800	1.0409
**Generator Voltages (Vg)**									
**V1**	1.0284	1.0138	1.0242	1.06	1.0395	1.0151	1.04	1.0170	1.088
**V2**	1.0044	0.9608	0.9953	1.0388	1.0259	0.9810	1.0298	0.9500	1.0805
**V3**	0.9844	1.0173	1.0098	1.0078	1.0077	1.0002	1.0099	1.0600	1.0825
**V6**	0.9872	0.9898	1.0176	0.9688	0.9982	1.0039	0.9776	1.0340	1.0802
**V8**	1.0262	1.0362	1.0268	0.9715	1.0158	1.0198	0.9855	1.0140	1.0862
**V9**	0.9834	1.0241	1.0283	0.9556	0.985	1.0254	0.9676	1.0600	1.0799
**V12**	0.9844	1.0136	1.0125	0.9891	0.9966	1.0081	0.9081	1.0090	1.0824
**Capacitor Banks (Qc)**									
**Qc18**	9	0.0463	0.0593	0	9.9846	0.0550	4	0.0063	4.8939
**Qc25**	7.0185	0.0590	0.0591	10	10	0.0590	15	0.1000	4.8324
**Qc53**	5.0387	0.0628	0.0382	9.5956	10	0.0381	11.678	0.0948	6.4528
**Ploss**	27.842	32.34	30.02	27.125	27.195	29.20	26.680	27.700	26.3956

**Table 14:** % Power loss reduction in standard 57-bus system with 25 decision variables.

Item	Base Case	PSO [75]	ICA [75]	ICA-PSO Hybrid [75]	FO-DPSO [6]	DSA [83]	FO-PSO with Entropy
**Ploss (MW)**	27.8637	27.842	27.125	27.195	26.680	27.700	26.395
**Loss reduction (%)**	-	0.077	2.651	2.399	4.248	0.587	5.268

The proposed technique is also tested for fitness function of voltage profile improvement of power system and outcomes of simulation depicts that proposed optimizer is more promising than other techniques which is duly stated in Table 15.

### 4.3. Comparative Study through Statistics

**Figure 12:**
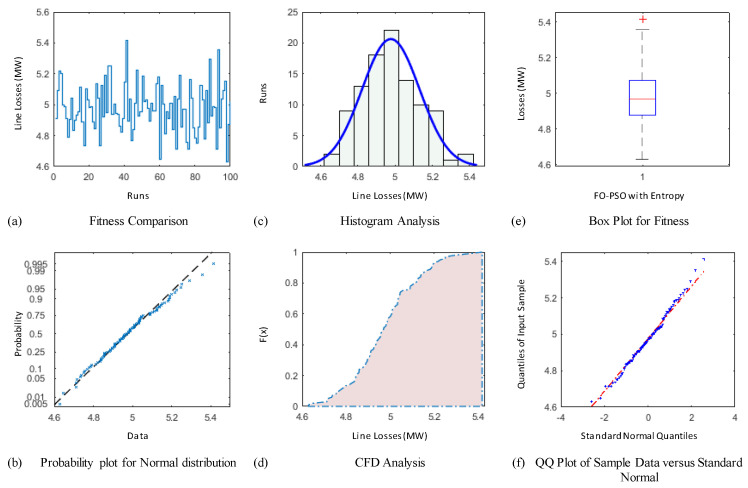
Statistical analysis of 30-bus system for power loss minimization with α = 0.3 based on (**a**) fitness comparison (**b**) probability plot (**c**) histogram analysis (**d**) CFD analysis (**e**) box plot representation (**f**) QQ plot.

**Figure 13:**
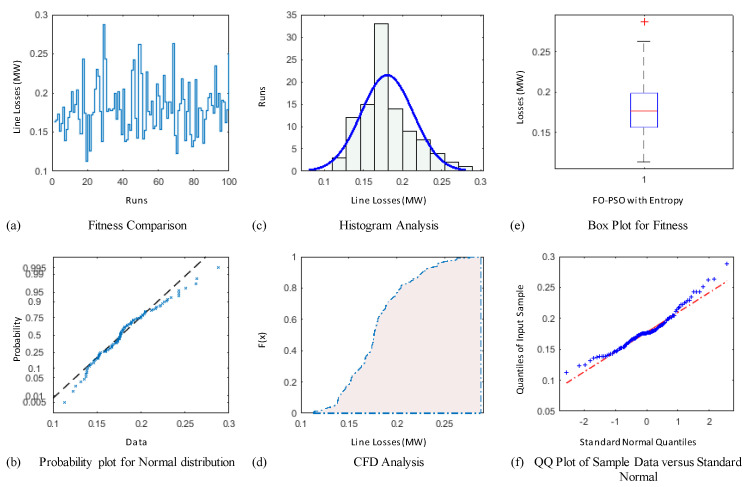
Statistical analysis of 30-bus system for voltage profile improvement with α = 0.4 based on (**a**) fitness comparison (**b**) probability plot (**c**) histogram analysis (**d**) CFD analysis (**e**) box plot representation (**f**) QQ plot.

**Figure 14:**
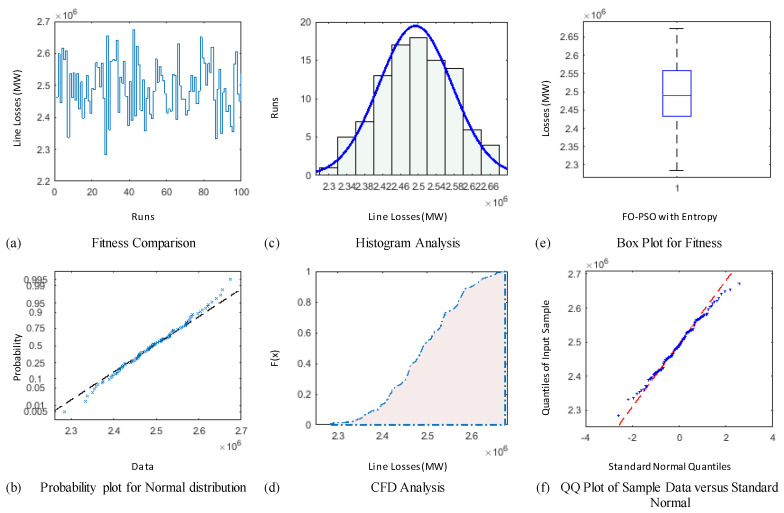
Statistical analysis of 30-bus system for operating cost minimization with α = 0.7 based on (**a**) fitness comparison (**b**) probability plot (**c**) histogram analysis (**d**) CFD analysis (**e**) box plot representation (**f**) QQ plot.

**Figure 15:**
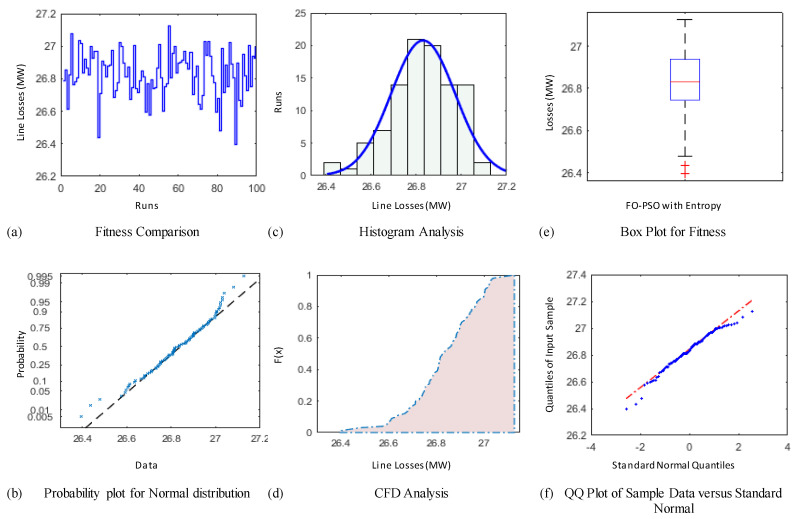
Statistical analysis of 57-bus system for power loss minimization with α = 0.9 based on (**a**) fitness comparison (**b**) probability plot (**c**) histogram analysis (**d**) CFD analysis (**e**) box plot representation (**f**) QQ plot.

**Figure 16:**
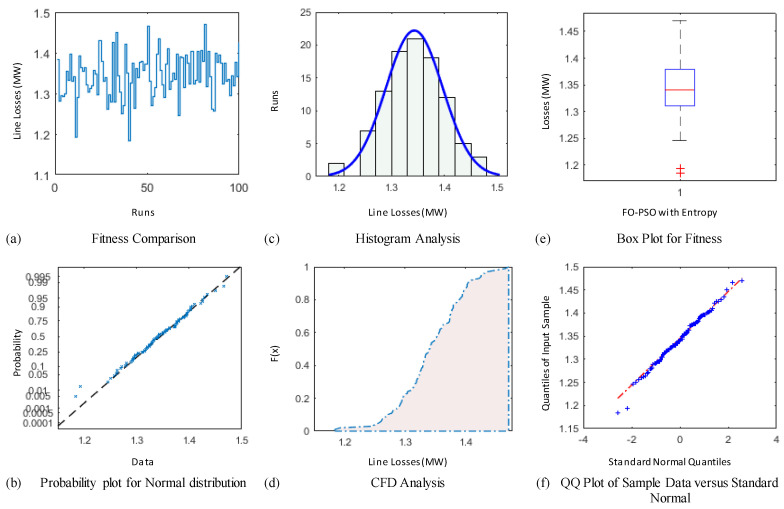
Statistical analysis of 57-bus system for voltage profile improvement with α = 0.3 based on (**a**) fitness comparison (**b**) probability plot (**c**) histogram analysis (**d**) CFD analysis (**e**) box plot representation (**f**) QQ plot.

**Figure 17:**
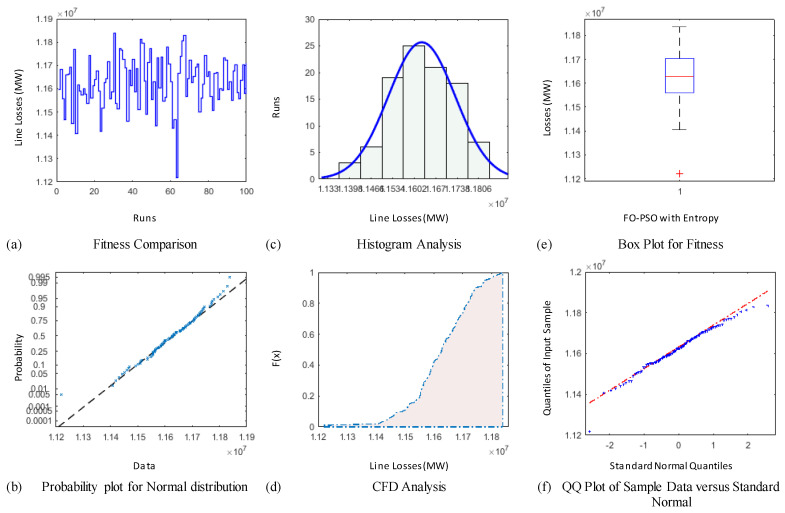
Statistical analysis of 57-bus system for operating cost minimization with α = 0.7 based on (**a**) fitness comparison (**b**) probability plot (**c**) histogram analysis (**d**) CFD analysis (**e**) box plot representation (**f**) QQ plot.

The demonstration of statistical illustrations are provided in this section to endorse consistent optimization inferences on account of designed optimization algorithm known as FO-PSO with entropy metric for optimal RPD problems. Therefore, the proposed optimization algorithm is executed for Hundred independent runs considering the best α for 30 and 57 Bus standard networks. The outcomes of statistical analysis, by means of minimum fitness evaluation function, probability charts of CDF, histogram studies, box plots, probability plot illustrations and quantile–quantile plot illustrations are presented in Figure 12, Figure 13, Figure 14, Figure 15, Figure 16 and Figure 17 for ORPD in standard electric networks.

In all case studies of optimal RPD presented in Figure 12a, Figure 13a, Figure 14a, Figure 15a, Figure 16a and Figure 17a show the minor distinction and also established reasonable accuracy and efficacy of FO-PSO with entropy metric optimization algorithm in each independent run. Furthermore, in Figure 12b, Figure 13b, Figure 14b, Figure 15b, Figure 16b and Figure 17b, probability charts demonstrate an ideal normal distribution of fitness values for all the cases which prove the precise optimization of the FO-PSO with entropy metric. The histogram illustrations presented in Figure 12c, Figure 13c, Figure 14c, Figure 15c, Figure 16c and Figure 17c for all ORPD scenarios illustrates that majority of the independent run of FO-PSO with entropy metric provide minimum gauges of $P_{loss}$. As provided in Figure 12d, Figure 13d, Figure 14d, Figure 15d, Figure 16d and Figure 17d the probability plots for CDF shows that 80% of the independent runs of proposed algorithm gives $P_{loss}$ inferior to 5.2 MW and 26.9 MW 30 and 57 bus networks, respectively. The outcomes of box plots in Figure 12e, Figure 13e, Figure 14e, Figure 15e, Figure 16e and Figure 17e for all cases of ORPD demonstrates that median of $P_{loss}$ is approximately 4.98 MW & 26.8 MW for 30 and 57 bus networks, respectively while the data spread is very close. The quantile–quantile plot presented in Figure 12f, Figure 13f, Figure 14f, Figure 15f, Figure 16f and Figure 17f show the ideal behavior of fitness versus the quantiles of a normal distribution for all cases of ORPD. All the above mentioned graphical illustrations of the statistical analysis carried for different ORPD scenarios demonstrate the efficacy, stability and robustness of proposed FO-PSO with entropy metric.

## 5. Conclusions

This paper proposed a new fractional swarm computing mechanism, namely, FO-PSO with entropy metric for the solution of ORPD problems for tuning the operational variables to reduce $P_{loss}$, overall cost while improving the VPI and meeting the consumer’s power demand. The designed strategy is viably tested on standard electric networks including IEEE 30 and 57 bus. The yielded outcomes from the FO-PSO with entropy metric are compared with the results reported in literature by the other well established algorithms where in all the given scenarios the proposed method demonstrate best performance by mean of evaluating least $P_{loss}$, overall cost and VPI in benchmark networks. Variants of FO-PSO with entropy metric are developed by adopting different fractional coefficients α=[0.1,0.2,…,0.9] and tested on both standard systems with considerable precision; however, the best performance of FO-PSO with entropy metric is achieved at α=0.3 for fitness function of power loss minimization, α=0.4 for voltage profile improvement and α=0.7 for operating cost minimization in case of 30-bus system. Similarly, in case of 57-bus system the best results of fitness functions are obtained at α=0.9, α=0.3 and α=0.7 for power loss minimization, voltage profile improvement and operating cost minimization, respectively. The efficacy of developed algorithms is endorsed through outcomes of statistics by means of the iterative updates depictions, cumulative distribution, histograms charts and quantile–quantile probability illustrations based on sixty independent trials of proposed algorithm adopting α=0.3 for all test systems which demonstrate the stable, robust and consistent nature of FO-PSO with entropy metric as an alternate, precise and promising computational technique. In the adopted scenarios, the developed algorithm has computed minimum losses, voltage deviation index, and overall cost in comparison with existing methods while adopting the same standard test system. In fact, fractional calculus has appeared as a mathematical tool to improve the performance of traditional PSO algorithm pertaining the main reason for its improved performance.

It should be noted that the better performance of the proposed FO-PSO with entropy metric is achieved for particular/selected cases of ORPD in terms of computing minimum value of objective functions compared to those, yielded/reported by other algorithms; however, there may be some situations in which other methods perform comparably or even marginally better. Indeed, theoretical analysis for a proper justification of the improved performance is always difficult, stiff, challenging, and complex task. Therefore, a stochastic process is adopted in the presented study for performance evaluation by means of statistical assessments based on Monte Carlo simulations, so, one should also investigate to provide theoretical analysis with proper mathematical justification. There are some situations with fractional orders α in [0-1] where the other methods perform comparably or even better; however, in the present study we have documented those situations with fractional orders only where the algorithm performs better than other algorithms in terms of computing minimum fitness evaluation function and suggested a solution for the readers and system operators.

In future one may design new variants of canonical algorithms based on synergy the of both the entropy and fractional calculus to improve the optimization characteristics and apply them to other problems of the energy sector.

## Figures and Tables

**Table 1:** Up-gradation of power system infrastructure.

Merits	De-Merits
Enhanced Power Evacuation Capacity	Cost (Capex & Opex)
Improved Load Management	Site Clearance & Security Issues
	Permits/Right of Way Issues

**Table 2:** Optimization of existing power system.

Merits	De-Merits
Minimize T&D losses	Marginal Improvement in System Capacity
Improvement of Voltage Profile Index (VPI)	
Enhanced System Stability	
Reduce Overall Cost of Operation	

**Table 3:** Load data.

Bus #	Load Detail (per unit)		Bus #	Load Detail (per unit)	
	**Q**	**P**		**Q**	**P**
B1	0.0000	0.0000	B16	0.0180	0.0350
B2	0.1270	0.2170	B17	0.0580	0.0900
B3	0.0120	0.0240	B18	0.0090	0.0320
B4	0.0160	0.0760	B19	0.0340	0.0950
B5	0.1900	0.9420	B20	0.0070	0.0220
B6	0.0000	0.0000	B21	0.1120	0.1750
B7	0.1090	0.2280	B22	0.0000	0.0000
B8	0.3000	0.3000	B23	0.0160	0.0320
B9	0.0000	0.0000	B24	0.0670	0.0870
B10	0.0200	0.0580	B25	0.0000	0.0000
B11	0.0000	0.0000	B26	0.0230	0.0350
B12	0.0750	0.1120	B27	0.0000	0.0000
B13	0.0000	0.0000	B28	0.0000	0.0000
B14	0.0160	0.0620	B29	0.0090	0.0240
B15	0.0250	0.0820	B30	0.0190	0.1060

**Table 4:** Transmission line data.

Transmission Line #	Line Impedance (per unit)		To Bus	From Bus
	X	R		
L1	0.0575	0.0192	2	1
L2	0.1852	0.0452	3	1
L3	0.1737	0.0570	4	2
L4	0.0379	0.0132	4	3
L5	0.1983	0.0472	5	2
L6	0.1763	0.0581	6	2
L7	0.0414	0.0119	6	4
L8	0.1160	0.0460	7	5
L9	0.0820	0.0267	7	6
L10	0.0420	0.0120	8	6
L11	0.2080	0.0000	9	6
L12	0.5560	0.0000	10	6
L13	0.2080	0.0000	11	9
L14	0.1100	0.0000	10	9
L15	0.2560	0.0000	12	4
L16	0.1400	0.0000	13	12
L17	0.2559	0.1231	14	12
L18	0.1304	0.0662	15	12
L19	0.1987	0.0945	16	12
L20	0.1997	0.2210	15	14
L21	0.1932	0.0824	17	16
L22	0.2185	0.1070	18	15
L23	0.1292	0.0639	19	18
L24	0.0680	0.0340	20	19
L25	0.2090	0.0936	20	10
L26	0.0845	0.0324	17	10
L27	0.0749	0.0348	21	10
L28	0.1499	0.0727	22	10
L29	0.0236	0.0116	22	21
L30	0.2020	0.1000	23	15
L31	0.1790	0.1150	24	22
L32	0.2700	0.1320	24	23
L33	0.3292	0.1885	25	24
L34	0.3800	0.2544	26	25
L35	0.2087	0.1093	27	25
L36	0.3960	0.0000	27	28
L37	0.4153	0.2198	29	27
L38	0.6027	0.3202	30	27
L39	0.4533	0.2399	30	29
L40	0.2000	0.6360	28	8
L41	0.0599	0.0169	28	6

**Table 5:** Generator bus data.

Bus #	Cost Coefficients		
	x	y	z
B1	0.0000	2.0000	0.003750
B2	0.0000	1.7500	0.017500
B5	0.0000	1.0000	0.062500
B8	0.0000	3.2500	0.008340
B11	0.0000	3.0000	0.025000
B13	0.0000	3.0000	0.025000

**Table 6:** Minimum and maximum limits for the decision variables.

	Decision Variables		
	Min	Max	Initial
T11	0.9000	1.1000	1.0780
T12	0.9000	1.1000	1.0690
T15	0.9000	1.1000	1.0320
T36	0.9000	1.1000	1.0680
P1	50.00	200.00	99.24
P2	20.00	80.00	80.00
P5	15.00	50.00	50.00
P8	10.00	35.00	20.00
P11	10.00	30.00	20.00
P13	12.00	40.00	20.00
V1	0.9500	1.1000	1.0500
V2	0.9500	1.1000	1.0400
V5	0.9500	1.1000	1.0100
V8	0.9500	1.1000	1.0100
V11	0.9500	1.1000	1.0500
V13	0.9500	1.1000	1.0500
Qx10	0.0000	5.0000	0.00
Qx12	0.0000	5.0000	0.00
Qx15	0.0000	5.0000	0.00
Qx17	0.0000	5.0000	0.0000
Qx20	0.0000	5.0000	0.0000
Qx21	0.0000	5.0000	0.0000
Qx23	0.0000	5.0000	0.0000
Qx24	0.0000	5.0000	0.0000
Qx29	0.0000	5.0000	0.0000
**Power losses (MW)**			5.8420
**Voltage deviations (VPI)**			1.16060
**Lmax**			0.21440

**Table 15:** Comparison of VPI for standard 57-bus system with 25 decision variables.

Item	Published Outcomes			Present
	NGBWCA [15]	WCA [15]	OGSA [15]	FO-PSO with Entropy
**VPI (p.u)**	1.2710	1.3852	1.1907	1.1844
